# Effect of Hybrid Type on Fermentation and Nutritional Parameters of Whole Plant Corn Silage

**DOI:** 10.3390/ani11061587

**Published:** 2021-05-28

**Authors:** Yue Liu, Guogen Wang, Hao Wu, Qingxiang Meng, Muhammad Zahoor Khan, Zhenming Zhou

**Affiliations:** State Key Laboratory of Animal Nutrition, College of Animal Science and Technology, China Agricultural University, Beijing 100193, China; yueliu@cau.edu.cn (Y.L.); wgy1341549373@163.com (G.W.); wu2213@cau.edu.cn (H.W.); qxmeng0624@126.com (Q.M.); zahoorcau@cau.edu.cn (M.Z.K.)

**Keywords:** hybrid type, dual-purpose corn, silage-specific corn, nutritive value, yield prediction and energy value, in situ digestibility

## Abstract

**Simple Summary:**

Two corn hybrid types, dual-purpose and silage-specific, were harvested at the one half to three fourths milk line and ensiled in fermentation bags for 60 days. Parameters such as fermentation quality, chemical composition, yield prediction and energy value, carbohydrate profile, and in situ digestibility were evaluated for the comparison of two corn types. Our analysis for the above parameters showed that under favourable production conditions, for whole-plant corn silage, the nutritive value per unit was higher for dual-purpose corn, but biomass yield and nutritive value per ha were higher in silage-specific corn. Our current study provides data for whole-plant corn silage research and reference data for ruminant nutrition in selecting corn silage.

**Abstract:**

This study was designed to evaluate the effect of hybrid type on the fermentation and nutritional parameters of whole-plant corn silage (dual-purpose and silage-specific corn). For this purpose, the two corn hybrid types were harvested at the one-half to three-fourths milk line and ensiled in fermentation bags (50 × 80 cm) for 60 day. Our results demonstrated that the ratio of lactic acid to acetic acid (*p* = 0.004), propionic acid (*p* < 0.001), Flieg point (*p* < 0.001), ether extract (*p* = 0.039), starch (*p* < 0.001), milk-per-ton index (*p* < 0.005), net energy for lactation (*p* = 0.003), total digestible nutrients (*p* < 0.001), neutral detergent soluble fiber (*p* =0.04), and in situ dry matter digestibility (TDMD_is_) (*p* < 0.001) were higher in dual-purpose corn silage, while the pH (*p* = 0.014), acetic acid (*p* = 0.007), the ratio of ammonia nitrogen to total nitrogen (*p* = 0.045), neutral detergent fiber (*p* < 0.001), acid detergent fiber (*p* < 0.001), acid detergent lignin (*p* < 0.001), dry matter yield per ha (*p* < 0.001), milk-per-acre index (*p* = 0.003), available neutral detergent fiber (*p* < 0.001), and unavailable neutral detergent fiber (*p* < 0.001) were higher in silage-specific corn silage. Based on our analysis, we concluded that under favourable production conditions for whole-plant corn silage, the nutritive value per unit was higher in dual-purpose corn while biomass yield and nutrient value per ha were higher in silage-specific corn.

## 1. Introduction

Corn (*Zea mays* L.) silage is an important fiber and energy source for ruminants [1]. Whole-plant corn silage (WPCS) has been widely used in ruminant nutrition to improve their production performance, e.g., dry matter intake [2], average daily gain [3], and milk yield [3,4]. With the progress of technology, corn yield has rapidly improved in China. The grain corn has been in surplus for the past ten years in China; however, the volume of imported forage has been increased [5]. Therefore, the Chinese government has encouraged the production of WPCS since 2015.

The effect of corn hybrid types on the quality of WPCS has been studied. Sheaffer et al. [6] compared the yield and quality among brown midrib (BMR), leafy (LFY), and conventional (CON) corn silage. Ferraretto et al. [7] reported the fermentation and digestibility of corn silage among BMR compared with dual-purpose or LFY. However, there are few comparative studies of WPCS from dual-purpose corn and LFY corn [8,9] and no study that compares WPCS from dual-purpose corn and silage-specific corn has been reported. Thomas et al. [8] demonstrated that dual-purpose and LFY corn silages have similar contents of fiber, starch, in vitro true dry matter disappearance (TDMD_vt_), in vitro NDF disappearance (NDFD_vt_), pH, and lactic acid concentration at similar content of DM. Similarly, Ballard et al. [9] reported a higher NDFD_vt_ for LFY than dual-purpose corn silage at similar maturities and moisture content. On the other hand, it has been reported that the quality of corn silage can vary due to differences in the quantity of grain and quality of stover present between hybrids [10,11]. Additionally, dual-purpose corn allows growers to harvest the crop as either grain or silage. Conversely, the silage-specific corn is characterized by tall plants and stay-green that contains a high forage yield and low proportion of grain components.

Therefore, the objective of this study was to evaluate the effect of hybrid types (dual-purpose and silage-specific corn) on fermentation quality, chemical composition, yield prediction and energy value, carbohydrate profile, and in situ digestibility of whole plant corn silage.

## 2. Materials and Methods

### 2.1. Ensiling of Whole-Plant Corn

The ensiling process of whole-plant corn silage hybrid types was conducted at the Beef Cattle Research Center at the China Agricultural University, Beijing. The dual-purpose and silage-specific corn hybrids were harvested in Tianjin and Shanxi at a seeding rate of 55,000 plants/ha and 65,000 plants/ha, respectively (Table 1). Corn was cut at the one-half and three-fourths milk lines on 29 August and 9 September 2016, respectively. Twenty plants of each variety were manually cut at approximately 10–15 cm above the ground. Four plants were harvested from each of five different sites within an experimental plot, resulting in twenty plants per corn hybrid. Fresh weights of individual plants were recorded, and then chopped using a hammer crusher (Changhong-330, Henan Xingyang Changheng Machinery Factory, Henan, China) with a theoretical cut length of 1–2 cm, and a 300 g sample from each hybrid was removed for DM analysis. The yield of each hybrid was calculated by including the contribution of the twenty individual plants. Thereafter, the chopped plants (5 kg, in triplicate) were immediately packed in fermentation bags with a one-way breathing valve (50 × 80 cm, Xinyu scarecrow agricultural park, Jiangxi, China) and vacuum sealed (DZ-2SE, Dongguan Qingye Packaging Machinery Ltd. Co., Guangdong, China). The plants were allowed to ferment at room temperature for 60 days. No silage inoculant was used during ensiling. The filling, compressing, and sealing processes were the same for both corn types.

### 2.2. Fermentation Quality

A subsample of 30 g (in triplicate) was weighed in a blender (FS-2, Changzhou Xinhang Instrument Factory, Jiangsu, China), diluted with 300 mL distilled water, homogenized for 2 min and passed through four layers of cheesecloth. The extracted solution’s pH was measured using an electrode (PHS-3C, Shanghai Leici Instrument Factory, Shanghai, China). The filtrate (20 mL) was stored at −20 °C for further analyses. The ammonia nitrogen (NH_3_-N) was determined according to the phenol–sodium hypochlorite colorimetric method (Broderick and Kang, 1980) using a spectrophotometer (UV-VIS 8500, Tianmei Scientific Instrument Co., Shanghai China), and the total nitrogen was determined using the Dumas combustion method (RaPid N III, Elementar, Germany). We analyzed lactic acid by ion chromatography (Dionex ICS-2500, Dionex instruments, California, CA, USA) equipped with an InoPac AS11-HC analysis column (4 × 250 mm), an InoPac AS11-HC protect column (4 × 50 mm), and an ASRS ULTRA II 4 mm suppressor. The sampling amount was 25 μL, the column temperature was 30 °C, the mobile phase was 50 mml/L sodium hydroxide solution and ultrapure water, and had a flow rate of 1 mL/min. VFA was analyzed by gas chromatography (SP-3420, Beijing Analytical Instrument Factory, Beijing, China) (Yan L and Qing X., 2006). The Flieg point was calculated using the following equation: Flieg point=220+(2×DM%)−15−(40×pH), and a Flieg point of 100–81, 80–61, 60–41, 40–21, and 20–0 represent excellent, good, medium, low, and poor silage quality, respectively [12].

### 2.3. Chemical Composition

The second subsample of 300 g was air-dried in a cool, ventilated place, and ground using a feed mill (DF-20, Wenling Linda machinery co. LTD, Zhejiang, China) to a particle size of 1 mm for further analyses. Then, samples were analyzed for DM (method 930.15), and ash (method 942.05) using the AOAC method [13]. Furthermore, the starch was analyzed using a total starch assay kit (Megazyme, Bray, Ireland; method 996.11) based on the AOAC method [13]. The nitrogen was analyzed using the Dumas combustion method (RaPid N III, Langenselbold, Germany). In addition, the CP was calculated using a 6.25 nitrogen-to-protein conversion factor. The ether extract (EE) was obtained using an automatic extractor (ANKOM XT101, ANKOM Technology Corp., Macedon, NY, USA). The determination of NDF, ADF, and acid detergent lignin (ADL) was performed according to the method reported by Van Soest et al. [14] and Robertson et al. [15] with heat-stable α-amylase, and performed using a fiber analyzer (ANKOM A220, ANKOM Technology Corp., Macedon, NY, USA). The water-soluble carbohydrate (WSC) was determined by anthrone–sulfuric acid colorimetry [16].

### 2.4. Yield Prediction and Energy Value

The DM yield was estimated according to the fresh weight per plant and the loss of DM ensiling. The milk-per-ton index (MT), milk-per-acre index (MA), and NE_L_ were calculated using the Milk 2006 model developed by the University of Wisconsin. The calculation of total digestible nutrients (TDN) was performed as reported by Weiss [17]. The composition was expressed on a DM basis (%).

$$\mathrm{TDN}(\%)=0.98\times (100-{\mathrm{NDF}}_{N}-\mathrm{CP}-\mathrm{Ash}-\mathrm{IADICP}\;-{\mathrm{EE})+K}_{\mathrm{dCP}}\times \mathrm{CP}+2.25\times (\mathrm{EE}-1)+{0.75\times (\mathrm{NDF}}_{N}\;-\mathrm{ADL})\times [1-{\left({\mathrm{ADL}/\mathrm{NDF}}_{N}\right)}^{0.667}]-7\;{\mathrm{NDF}}_{N}=\mathrm{NDF}-\mathrm{NDICP}+\mathrm{IADICP}\;\mathrm{IADICP}=0.070\times \mathrm{ADICP}\;K_{\mathrm{dCP}}=\exp(-0.0012\times \mathrm{ADICP})$$

### 2.5. Carbohydrate Profile

The carbohydrate composition profile (except unavailable neutral detergent fiber, **CC**) was calculated according to the equations as described by NRC (2016) [18], CC was calculated according to Sniffen et al. [19]. The composition was expressed on a DM basis (%).

Non-neutral detergent fiber (non-NDF)=100−CP−NDF−EE−Ash

Organic acid (OA)=lactic acid+acetic acid+propionic acid+butyric acid+other OA

Water-soluble carbohydrate (CA)=WSC

CB1=starch

Neutral detergent soluble fiber (CB2)=non-NDF−OA−CA−CB1

Unavailable neutral detergent fiber (CC)=2.4×ADL

Available neutral detergent fiber (CB3)=NDF−CC

### 2.6. In Situ Digestibility

The digestibility of corn silage was determined by the in situ nylon bag technique reported by Fei et al. [20]. Three Angus steers (380 ± 15 kg live weight) fitted with permanent rumen cannulas (Beef Cattle Research Center, China Agricultural University, Beijing, China) were used in this study, and the animals were approved by the China Agricultural University Animal Care and Use Committee (AW06059102-2, Beijing, China). The animals were fed a total mixed ration at 8:00 and 17:00 according to NRC (2000) [21] and had ad libitum access to drinking water and a mineral block. Each sample (4.00 ± 0.01 g) was transferred to a single nylon bag (80 × 140 mm; Beef Cattle Research Center, China Agricultural University) with a pore size of 37 µm. Each type of corn silage had nine nylon bags, three nylon bags placed in each cattle, and then samples were incubated in the rumen for 24 or 48 h. Following their removal, the bags were immediately rinsed in cold water and washed 6 times (1 min/rinse) in a washing machine until the water became clear and then dried at 105 °C for 48 h for in situ dry matter digestibility (TDMD_is_) analysis, and dried at 65 °C for 48 h for in situ neutral detergent fiber digestibility (NDFD_is_) analysis.

### 2.7. Statistical Analysis

The statistical analysis was performed using SAS 9.0 software (SAS Institute Inc., Cary, NC, USA), and a two-tailed Student’s t-test [22] was used for comparison between the two corn types. A significant difference was considered as *p* < 0.05.

## 3. Results

### 3.1. Fermentation Parameters

The fermentation parameters of dual-purpose and silage-specific corn silage are presented in Table 2. Our results demonstrated that the dual-purpose corn silage contained more propionic acid (*p* < 0.001), had a higher Flieg point (*p* < 0.001), and the ratio of lactic acid to acetic acid (*p* = 0.004) was also higher than the silage-specific corn silage. Consistently, the silage-specific corn silage contained higher acetic acid (*p* = 0.007), pH (*p* = 0.014), and ratio of ammonia nitrogen to total nitrogen (*p* = 0.045). Additionally, following 60 d of fermentation, pH values ranged between 3.84 and 3.88. The ratio of lactic acid to acetic acid was >3:1, while no butyric acid was detected. Finally, we also documented that the Flieg points were greater than 108.00 points in our current experiment.

### 3.2. Chemical Composition

There were significant differences (*p* < 0.01) in the DM, starch, NDF, ADF, and ADL between the two corn silages, as mentioned in Table 3. Consistently, our data reported a significant difference for EE (*p* = 0.039) between the dual-purpose and silage-specific corn silages. Consequently, our results have shown that the percentage compositions of starch and EE were higher in the dual-purpose silage than in the silage-specific corn silage. In contrast, the NDF, ADF, and ADL were higher in the silage-specific corn silage in comparison to the dual-purpose corn silage (Table 3).

### 3.3. Yield Production and Energy Value

Yield production and energy value of the dual-purpose and silage-specific corn silages are summarized in Table 4. Our findings showed that the significant difference (*p* < 0.001) in DM yield, MA, TDN, MT (*p* = 0.005), and NE_L_ (*p* = 0.003). In addition, the DM yield and MA were higher in silage-specific corn silage, but the MT, NE_L_, and TDN were lower compared to the dual-purpose corn silage.

### 3.4. Carbohydrate Profile

As presented in Table 5, the significant differences (*p* < 0.001) for CB1, CB3, CC, and CB2 (*p* = 0.04) were recorded between the dual-purpose and silage-specific corn silages. In addition, the CB1 and CB2 were higher (*p* < 0.05) in the dual-purpose corn silage, whereas the CB3 and CC were higher (*p* < 0.001) in the silage-specific corn silage.

### 3.5. In Situ Digestibility

Our data showed a significant difference (*p* < 0.001) for in situ DM digestibility (Table 6). Specifically, the TDMD_is_ at 24 h (55.21%) and 48 h (71.82%) in the dual-purpose corn silage was comparatively higher than that of the silage-specific corn silage at 24 h (47.33%) and 48 h (61.57%). However, no difference was reported for the NDFD_is_ at 24 h and 48 h between the two corn silages.

## 4. Discussion

This experiment was designed based on the actual production of dual-purpose corn and silage-specific corn with samples collected from actual corn production. A large number of corn hybrid varieties were collected for two corn types, a good representation for each type of corn. To compare the dual-purpose and silage-specific corn silages in this study, we harvested two types of corn at the same stage. After analyzing, we observed a good fermentation quality between the two corn silages. Moreover, the dual-purpose corn silage has a higher ratio of lactic acid to acetic acid, propionic acid, Flieg point, EE, starch, MT, TDN, NE_L_, CB2, and TDMD_is_, and lower pH, acetic acid, NH_3_-N/TN, NDF, ADF, ADL, DM yield per ha, MA, CB2, and CB3 compared to the silage-specific corn silage.

A lower pH value is usually an indicator of increased lactic acid concentration, thereby implying better fermentation of silages during the ensiling period; our results both fall into the 3.80 to 4.20 range [24]. The acetic acid in silage results from the fermentation of sugar by heterofermentative lactic bacteria and intestinal bacteria [25], which is the most abundant organic acid produced by ideal fermentation; our results were within the recommended value (4–6%) [18], similar to Nennich et al. [26], suggesting the corn was well fermented in this experiment. The lower ratio of lactic acid to acetic acid [18] also suggests ideal fermentation conditions in the present study. Additionally, the NH_3_-N/TN indicates the degradation of CP, which is far below 10%, suggesting that both corn silges were well fermented in the current study [3]. The butyric acid was not detected in this experiment, which is in line with the findings reported by Zhou et al. [27]. They argued that butyric acid concentration was low and biologically negligible in whole-plant corn silage. Therefore, for two corn silages, the higher Flieg points with excellent fermentation quality [12] might be due to being well vacuum-sealed. Brüning et al. [28] had reported that low compaction and delayed sealing adversely impacts silage quality.

Shaver et al. [29] reported that CON corn (34.30%) has 6.5% more DM content than BMR corn (32.20%) at the one-half milk line, whereas Johnson et al. [30] found that a 7.70% difference of corn silage in DM content at the two-thirds milk line. In our findings, dual-purpose corn silage had 3.97% more DM content than silage-specific corn silage at the one-half to three-fourths milk lines (33.34% vs. 29.37%, respectively). The difference in the DM between the two hybrid types might be ascribed to the different proportion of different parts of the plant: silage-specific corn has a higher proportion of leaves, stalks, and is lower in grain content than dual-purpose corn [8]. Xu et al. [31] also observed that the different plant parts of corn silage had an impact on the DM content and the proportion of cob and grain was significantly higher in dual-purpose corn than that of LFY corn, while the opposite results were obtained for leaves and stalks. A study reported a significant difference in the NDF and ADF between a leafy-nutridense corn silage and a yellow-dent corn silage [32]. It has been demonstrated that the differences in nutritional parameters may be because of the different parts of the plant with varying proportions of fiber [31]. It has been well studied that the starch is mainly present in the grain; thus, in our current study, we also reported a higher starch content in the dual-purpose corn silage compared to the silage-specific corn silage. Accordingly, a study also documented that the starch content was higher in CON hybrids than in high-biomass hybrids at three different harvest times [33]. In the future, studies may provide more information on selecting different harvest times with different corn hybrid types, when compared to chemical composition.

In our current study, we found higher MT and energy values in dual-purpose corn silage, but a lower DM yield per ha and MA in silage-specific corn silage, which might be due to the higher nutritive value per unit in dual-purpose corn silage and the higher biomass yield and nutritive value per ha in silage-specific corn silage. The carbohydrate compositions were divided into six fractions: OA, CA, CB1, CB2, CB3, and CC. [18]. The OA is the acid produced by fermentation and the organic acid that remained in raw materials, which is considered to be 100% digestible [34]. The CA is fully utilized in the rumen, while the CB1 is partially degraded and the CB3 is 20% digestible [35]. The CB2 showed a rapid degradation (20% to 40% per hour), while the CC was not digestible in the rumen, small intestine, or posterior intestine [18]. The higher contents of CB1 and CB2 in the dual-purpose corn silage and the higher contents of CB3 and CC in the silage-specific corn silage suggest that the nutrient degradation rate of rumen for the dual-purpose corn silage was lower than the rumen degradation rate of silage-specific corn silage.

The 24 h TDMD_is_ values in our study were similar to the result (53%, 2/3 milk line) obtained by Shaver et al. [29]. However, the 24 h NDFD_is_ values reported in the current study were much higher than the documented results of Shaver et al. [29]. In addition, the 48 h NDFD_is_ values were higher than the 30 h NDFD_is_ of Akins and Shaver [4]. These differences are mainly ascribed to the different corn hybrids with different characteristics and different harvest times. In this experiment, the 24 h and 48 h DMD_is_ and NDFD_is_ were higher in the dual-purpose corn, while the ADL was higher in the silage-specific corn. This might be because of a high content of ADL that inhibited both the DMD_is_ [36] and NDFD_is_ [18]. Additionally, the 48 h DMD_is_ was higher than the 24 h DMD_is_, suggesting that some nutrients may not be adequately digested following 24 h of fermentation.

The silage-specific corn with tall plants has less resistance to wind as compared to the dual-purpose corn. However, the necessary conditions for plant growth were favourable in our study, and no strong wind effect was noticed until harvesting. However, strong winds and an insufficient supply of water and fertilizer will lead to a serious lodging condition, and under these conditions, the yield of nutritive value per ha cannot be expected to be higher in silage-specific corn. Therefore, the specific weather conditions should be combined with the region to carefully choose corn for planting.

## 5. Conclusions

Altogether, we concluded that corn hybrid type had significant effects on the fermentation quality, chemical composition, yield production and energy value, carbohydrate profile, and in situ digestibility of whole-plant silage. Although the fermentation quality of the two corn hybrid types was good, comparatively, the ratio of lactic acid to acetic acid, propionic acid, Flieg point, EE, starch, MA, CB1, and DMD_is_ of the dual-purpose corn silage were higher than in the silage-specific corn, while the NDF, ADF, ADL, DM yield per ha, MT, CB2, and CC were higher in the silage-specific corn silage. Thus, in the present study, we concluded that under favourable production conditions the nutritive value per unit is higher in dual-purpose corn silage, but the biomass yield and nutritive value per ha were higher in the silage-specific corn silage. In the future, feeding experiments are needed to verify this conclusion in combination with production performance and animal health status.

## Figures and Tables

**Table 1:** Information on types and varieties of corn.

Corn Types	Varieties	Location	Plants/ha
Dual-purpose corn (19 corn varieties)	Luyu 36, Wofeng 9, Zhengdan 958, Jingke 25, Jiyuan 128, Derun 98, Jundan 128, Jiudan 100, Jiudan 25, Wurui 605, Dongyu 108, Jiudan 57, Xianfeng 32D22, Weifeng 6, Xinyu 35, Huanong 866, Songyu 656, Jindan 52, Jundan 20	Tianjin	55,000
Silage-specific corn (13 corn varieties)	Yu Silage 23, Zheng Silage 1, Dafeng Silage 1, Beinong Silage 356, QS5, V80, HC45, QS3, Dafengsiyu 12, HC46, Zhongbei Silage 410, Jinling Silage 17, Jingke Silage 516	Shanxi	65,000

**Table 2:** Fermentation parameters of dual-purpose and silage-specific corn silage.

Item ^1^	Dual-Purpose Corn	Silage-Specific Corn	SEM ^3^	*p*-Value
pH	3.84	3.88	0.01	0.014
Lactic acid, %	4.86	5.06	0.31	0.640
Acetic acid, %	1.02	1.38	0.08	0.007
Propionic acid, %	0.12	0.03	0.01	<0.001
Lactic acid:acetic acid	5.25	3.67	0.33	0.004
Butyric acid, %	ND ^2^	ND	-	-
NH_3_-N/TN, %	3.73	4.39	0.20	0.045
Flieg point ^3^	117.95	108.60	1.80	<0.001

^1^ Lactic acid: Acetic acid = the ratio of lactic acid and acetic acid; NH_3_N/TN = the ratio of ammonia nitrogen to total nitrogen; ^2^ ND = not detected; ^3^ $\mathrm{Flieg}\;\mathrm{point}=220\;+\;\left(2\times \mathrm{DM}\%-15\right)-\left(40\times \mathrm{pH}\right);$ ^3^ SEM: standard error of the mean.

**Table 3:** Chemical compositions of the dual-purpose and the silage-specific corn silage (DM basis).

Item ^1^	Dual-Purpose Corn	Silage-Specific Corn	SEM ^2^	*p*-Value
DM, %	33.34	29.37	1.04	0.009
CP, %	8.73	8.82	0.15	0.702
EE, %	2.38	2.08	0.10	0.039
Starch, %	27.75	22.90	0.78	<0.001
WSC, %	2.26	2.29	0.19	0.323
NDF, %	43.12	50.34	0.71	<0.001
ADF, %	22.49	25.97	0.45	<0.001
ADL, %	2.47	3.14	0.10	<0.001
Ash, %	4.90	4.88	0.21	0.951

^1^ DM = dry matter; CP = crude protein; EE = ether extract; WSC = water soluble carbohydrate; NDF = neutral detergent fiber; ADF = acid detergent fiber; ADL = acid detergent lignin; ^2^ SEM: standard error of the mean.

**Table 4:** Yield prediction and energy values of the dual-purpose and silage-specific corn silages.

Item ^1^	Dual-Purpose Corn	Silage-Specific Corn	SEM ^2^	*p*-Value
DM yield, ton/ha	13.35	21.51	0.751	<0.001
MT, ton/ton	1.26	1.15	0.027	0.005
MA, ton/ha	16.79	24.42	0.704	<0.001
NE_L_, MJ/kg	0.33	0.31	0.004	0.003
TDN	71.84	68.36	0.511	<0.001

^1^ DM yield = dry matter yield; MT = milk-per-ton index; MA = milk-per-acre index; NE_L_ = net energy for lactation; TDN = total digestible nutrients; ^2^ SEM: standard error of the mean.

**Table 5:** Carbohydrate profile of the dual-purpose and silage-specific corn silages (DM basis).

Item ^1^	Dual-Purpose Corn	Silage-Specific Corn	SEM ^2^	*p*-Value
OA, %	5.99	6.46	0.35	0.332
CA, %	2.26	2.29	0.19	0.923
CB1, %	27.75	22.90	0.78	<0.001
CB2, %	4.91	2.94	0.66	0.040
CB3, %	37.18	42.80	0.63	<0.001
CC, %	5.94	7.54	0.23	<0.001

^1^ OA = organic acid; WSC = water soluble carbohydrate; CB1 = starch; CB2 = neutral detergent soluble fiber; CB3 = available neutral detergent fiber; CC = unavailable neutral detergent fiber; ^2^ SEM: standard error of the mean.

**Table 6:** In situ digestibility of the dual-purpose and silage-specific corn silages (DM basis).

Item ^1^	Dual-Purpose Corn (%)	Silage-Specific Corn (%)	SEM ^2^	*p*-Value
24 h TDMD_is_	55.21	47.33	0.89	<0.001
48 h TDMD_is_	71.68	61.57	0.75	<0.001
24 h NDFD_is_	21.57	22.01	0.63	0.634
48 h NDFD_is_	46.87	44.74	0.89	0.102

^1^ TDMD_is_ = in situ dry matter digestibility; NDFD_is_ = in situ neutral detergent fiber digestibility [23]; ^2^ SEM: standard error of the mean.

## Data Availability

All data are presented in the text and tables of this manuscript.
